# Maternal Risk Factors and Neonatal Outcome of the Admitted Patients for Preterm Spontaneous Uterine Contractions

**Published:** 2011-12-01

**Authors:** B Namavar Jahromi, L Salarian, Z Shiravani

**Affiliations:** 1 Department of Obstetrics and Gynecology, Perinatology Research Center, Shiraz University of Medical Sciences, Shiraz, Iran

**Keywords:** Preterm birth, Preterm uterine contraction, Preterm labor, Risk factor, Abortion, Infertility

## Abstract

**Background:**

Preterm birth (PTB) is one of the most important unsolved problems in reproductive medicine. This study aims to evaluate several maternal risk factors and outcome of pregnancies who were admitted for preterm spontaneous uterine contractions (PSUC).

**Methods:**

From September 2007 to February 2009, 327 cases who were admitted for PSUC were retrospectively studied. They were classified according to their fetal numbers and presence of true versus threatened preterm labor (PTL).

**Results:**

There were 297 (90.82%) singleton, 27 (8.25%) twin and 3 (0.91%) triplet pregnancies. Only 12 women (3.6%) fulfilled the ACOG criteria for PTL who delivered in a few hours and 315 cases (96.3%) were classified as threatened PTL and most of them were discharged undelivered from the hospital. 103 cases were missed and 224 mothers and their 247 neonates remained. 121 women from this cohort had PTB and delivered before 259 days (54%). Pregnancy outcomes including; the time interval between admission for PSUC and delivery, the mean gestational ages at birth, birth weights, number and duration of NICU admissions were evaluated in each group.

**Conclusion:**

Regular uterine contractions even in the absence of cervical changes should be considered as a potential risk factor for PTB. The most frequently associated maternal risk factors were history of abortion, infertility and previous PTB, and the most frequently associated complications were preterm rupture of membranes, vaginal bleeding and febrile diseases.

## Introduction

Preterm birth (PTB) is one of the most important unsolved problems in reproductive medicine. PTB is defined as delivery that occurs at more than 20 and less than 37 gestational weeks. PTB is the leading cause of perinatal morbidity and neonatal mortality[1][2]. The incidence of PTB in most developed countries has remained constant in over the past 3 decades at about 5-10% [3]. However; data demonstrates a steady rise in the preterm birth rate in certain populations over the past 20 years[1].

Spontaneous PTB occurs when the parturitional process begins in the absence of overt maternal or fetal illness[2]. Decreased local progestrone concentrations, oxytocin initiation, decidual activation[4] and deviation from normal fetal growth[5] had been implicated as pathogenesis of PTB.

On the other hand, preterm labor (PTL) which is the onset of regular uterine contractions accompanying with cervical change, after the 20th week of gestation and before 37 completed weeks or 259 days of pregnancy [1],[2] proceeds PTB. However, outcome of threatened PTL which is diagnosed when there are documented uterine contractions without evidence of cervical changes[3] is not yet accurately documented.

In this study, we retrospectively followed the women who were admitted for preterm spontaneous uterine contractions (PSUC) and several maternal risk factors, complications and neonatal outcomes were evaluated.

## Materials and Methods

All of the 327 pregnant women who were admitted for PSUC in Hafez Hospital from 23rd September 2007 to 28th February 2009 were included in this study. Data were collected retrospectively from the admission charts. PTL was confirmed for every patient according to ACOG criteria if the patient had 4 contractions in 20 minutes or 8 in 60 minutes plus progressive changes in the cervix and cervical dilatation greater than 1 cm and cervical effacement of 80 percent or greater[1]. However, the patients with documented PSUC without cervical changes were considered to have threatened PTL[3]. Gestational ages were calculated from the first day of the last menstrual period and confirmed by ultrasound scan and if it was less than 259 days, the pregnancy was considered to be preterm. The patients were managed according to the standard protocols after admission[6].

Simultaneous maternal risk factors such as history of previous PTB, abortion, infertility, infectious diseases, uterine diseases, endocrine or any organ system diseases were evaluated. Data about pregnancy complications such as rupture of membranes (ROM), vaginal bleeding, poly- or oligohydramnious, diabetes, hypertensive disorders were extracted from the hospital charts. The cases were followed up to delivery and the time interval between admission for PSUC and birth, their neonatal outcomes like birth weight, gestational age at delivery, number and duration of NICU admissions, congenital anomalies and neonatal deaths were also evaluated. The cases were classified according to their fetal numbers and studied separately. This study was approved by Shiraz University of Medical Sciences Review Board. Statistical analysis was performed by SPSS software (Version 16, Chicago, IL, USA) using Kruskal-Wallis, Mann-Whitney U and Fisher's exact tests for analysis and p<0.05 was considered significant.

## Results

During September 2007 and February 2009, 6124 deliveries occurred in Hafez Hospital and there were 327 pregnant women who were admitted for the management of spontaneous preterm labor during this time period. Among the 327 women who entered this study, there were 297 (90.82%) singleton, 27 (8.25%) twin and 3 (0.91%) triplet pregnancies. The mean maternal age was 26±5.16 years for the singleton group, 25.2±4.6 years for the twin group and 24.6±4.0 years for the triplet group respectively (p=0.4). The mean gestational age at the admission time was 230±24.6 days for the singleton group, 228± 2.9 days for the twin group and 225.6± 11.5 days for the triplet group ( p=0.5).

Simultaneous maternal diseases and the obstetrical complications of these women are presented in Table 1 and Table 2. Nine singleton women (3%) out of 297 were considered to have true PTL based on ACOG criteria and delivered in 59.2±131.7 hours from the admission time. However, 288 singletons (97%) were categorized in threatened PTL group without any progressive cervical changes. In the twin group, two women (7.4 %) with true PTL delivered in 1.25±1.06 hours and 25 twin cases (92.5 %) had threatened PTL. However, from the 3 triplet cases, one woman with true PTL delivered after 0.5 hour from admission and 2 others had threatened PTL.

We found that only 12 women (3.6%) fulfilled the ACOG criteria for PTL and 315 cases (96.3%) were classified as threatened PTL. From the 315 undelivered cases that most of them were discharged from the hospital, 103 women did not come back to deliver in Hafez Hospital and were missed. So overall, 224 mothers and their 247 neonates remained for the final analysis. We found that 121 women from this cohort finally had PTB before 259 days (54%). Considering the fact that totally 6124 deliveries occurred during the study period, the rate of spontaneous PTB following PTL or threatened PTL was found to be 1.97% in this center. Characteristics of the pregnancies which were followed up to delivery are presented in Table 3.

**Fig. 1:**
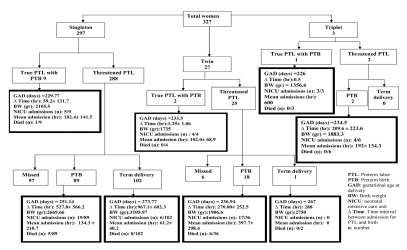
Outcome of pregnancies who were admitted for preterm spontaneous uterine contractions.

Eighty nine women from the singleton group, eighty from the twin group and two women from the triplet group finally had PTB. Figure 1 shows the outcome of pregnancies who were admitted for PSUC in detail. Examination of the neonates after birth showed that 5 singleton babies (2.5%) had anomalies as hydrops 1 (0.5%), hypospedias 1 (0.5%), polydactily 1 (0.5%), and club foot 2 (1%). However 6 neonates (15.7%) from the twin group had anomalies including hydrops 1 (2.63%), Down syndrome 1 (2.63%), anomalies of the face and extremities 2 (5.2%) and heart disease 2 (5.2%). Three neonates from the triplet group (33.3%) had anomalies including heart disease 2 (22.2%) and bowel obstruction 1 (11.1%).

**Table 1:** Simultaneous maternal diseases in the women with Preterm spontaneous uterine contractions at the admission time

**Maternal diseases**	**Singleton no. (%) n=297**	**Twin no. (%) n=27**	**Triplet no. (%) n=3**	**P value^a^**
History of at least one previous abortion	65 (21.8)	9 (33)	1 (33)	0.297
Infertility	49 (16.49)	10 (37)	2 (66.6)	0.227
Primary infertility	33 (11.1)	7 (25.9)	2 (66.6)	0.004
Secondary infertility	16 (5.38)	3 (11.1)	0	0.006
History of Previous PTB	26 (8.75)	1 (3.7)	1 (33)	0.001
Uterine diseases	25 (8.41)	2 (7.4)	0	0.001
Cervical incompetence	13 (4.37)	2 (7.4)	0	0.001
Myoma in uterus	8 (2.69)	0	0	-
Bicornate uterus	3 (1.01)	0	0	-
Septated uterus	1 (0.33)	0	0	-
Cardiac diseases	19 (6.39)	1 (3.7)	0	0.001
Mitral valve Prolapse	16 (5.38)	0	0	-
Aortic stenosis	1 (0.33)	0	0	-
Subaortic web	1 (0.33)	0	0	-
Pulmonary HTN	1 (0.33)	1 (3.7)	0	0.001
Symptomatic renal stone	17 (5.72)	0	0	-
tdyroid disease	16 (5.38)	3 (11.10	0	0.001
Hypotdyroidism	10 (3.36)	1 (3.7)	0	0.001
Hypertdyroidism	3 (1.01)	2 (7.4)	0	0.001
Chronic HTN	9 (3.03)	0	0	-
Astdma	4 (1.34)	0	0	-
Antiphospholipid antibody syndrome	4 (1.34)	2 (7.4)	0	0.001
Overt Diabetes	2 (0.67)	0	0	-

^a^ P value was computed if the sample size was sufficient for Fisher's exact test to evaluate the association between presence of the certain conditions and number of fetuses.

**Table 2:** Pregnancy complications of the women with preterm spontaneous uterine contractions

**Complications**	**Singleton no. (%) n=297**	**Twin no. (%) n=27**	**Triplet no. (%) n=3**	**P value a**
Preterm ROM	52 (17.5)	12 (44.4)	2 (66.6)	0.001
Vaginal bleeding	31 (10.43)	4 (14.8)	0	0.001
Abruption	4 (1.34)	1 (3.7)	0	0.001
Placenta Previa	3 (1)	1 (3.7)	0	0.001
Pyelonephritis	27 (9.1)	1 (3.7)	0	0.001
Pneumonia	3 (1)	1 (3.7)	0	0.001
Hypertensive disorders	20 (6.73)	2 (7.4)	0	0.001
Gestational Diabetes	20 (6.73)	3 (11.1)	0	0.001
Polyhydramnious	6 (2.02)	1 (3.7)	0	0.001
Oligohydramnious	6 (2.02)	1 (3.7)	0	0.001

^a^ The triplet group was excluded from the analysis if the complication was absent in the group.

**Table 3:** Pregnancy outcome of the women with preterm spontaneous uterine contractions who were followed up to delivery (The data are presented as n (%) or mean± SD)

		**Group 1 singletons n=200**	**Group 2 twins n=21**		**Group 3 triplets n=3**	**P value**
Mean gestational age at delivery (days)		261.7±20.1	238±24.27		231.6±7.37	<0.001^a^
Mean time interval between admission and delivery( hours)		730.0±628.2	245.4±246.5		139.9±198.9	<0.001^a^
Preterm delivery <259 days	98 (49)			20 (95.2)	3 (100)	0.001
Cesarean delivery	106 (53)			18 (85.7)	3 (100)	0.003
Vaginal delivery	94 (47)			3 (14.28)	0	0.003
Mean birtd weight (gr)	2855.2±655.37			1947.89±670.7	1707.7±420.8	<0.001^a^
Female	98 (49)			14 (33.33)	5 (55.5)	0.149
Neonates who had NICU admission	30 (15)			17 (44.7)	7 (77.7)	<0.001
Mean of NICU admitted hours	127.9±178.9			290.2±277.7	366.9±251.9	<0.001^a^
Deatd	6 (3)			4 (3)	0	0.147

^a^ The mean difference is significant between group (1, 2) and (1, 3)

## Discussion

In this retrospective study, we included all of 327 cases who were admitted to Hafez Hospital for spontaneous uterine contractions from 23rd September 2007 to 28th February 2009. The characteristics of the pelvic examinations and contraction frequencies of these cases on the admission time were compared with ACOG criteria to confirm the diagnosis of PTL[1]. Surprisingly, we found that only 12 women (3.6%) fulfilled the criteria and 315 cases (96.3%) were classified as threatened PLP.3 We were able to follow 224 of these women up to the time of delivery and found that 121 (54%) women had preterm deliveries before 259 days.

Considering the time interval between admission and delivery in the true PLP group showed that ACOG criteria identified the women which were truly in labor and usually no therapeutic modality was able to stop their preterm deliveries. However, most of the women (109/224) in the threatened PTL group (48.6%) had finally PTB even though the time interval between admission and delivery was longer. So presence of PSUC should be considered as a potent risk factor for PTB even if cervical dilatation has not yet occurred in the early stages. It was previously reported that contraction frequency is significantly greater in the women with preterm deliveries[7].

There are published data showing that in addition to spontaneous PTB, 20% to 30% of PTB are considered medically indicated to avoid or minimize maternal and/or fetal complications. Advances in maternal, fetal, and neonatal management have led to an increased willingness to deliver high-risk pregnancies preterm[8][9]. During this study period, 6124 delivery occurred in this center. So the rate of PTB following spontaneous PTL was calculated to be 1.97%. It should be noticed that during this time period, other PTBs occurred in this center due to maternal or fetal indications which were not included in this study and this data shows only the spontaneous PTB rate and not the total. Considering the fact that 40-45% of PTBs follow spontaneous labor[4], the rate of total PTBs in this center can roughly be estimated to be about 5% during the time period.

Multifetal pregnancies are at greater risks for preterm deliveries and increasing the fetal numbers increases the risk[10]. We classified the cases on the base of their fetal numbers and evaluated the groups separately. Following the cases to the time of delivery showed that finally PTB happened in 49% (98/200) of singletons, 95.23% (20/21) of twins and 100% (3/3) of triplets who presented with threatened PTL as shown in Figure 1.

Occurrence of at least one previous spontaneous abortion was evaluated among the three groups which was 21.8%( 65/297) for singleton, 33% (9/27) for twin and 33% (1/3) for the triplet group. Suggesting that history of at least one previous spontaneous abortion can be considered as a risk factor for development of PTL in the subsequent pregnancies. However association of first trimester abortions and PTB is still controversial but there are systematic reviews showing that induced and spontaneous abortions were associated with increased risk of PTB in subsequent pregnancies[11][12]. The results of this study may support the hypothesis that pregnancy loss and PTB may have unique etiologies[13].

We noticed a high percentage of women who received infertility treatments of 16.49%, 37% and 66.6% among singleton, twin and triplet pregnancies, respectively. A high number of preterm multiple gestations associated with assisted reproductive technologies as an important contributor to preterm deliveries was documented. However, it is reported that even singleton pregnancies after IVF and infertility treatments are at increased risk of PTB[14][15]. Our findings also suggested that women with infertility should be considered as a high risk population for PTL.

We found that 8.75% (26/297) of women in the singleton group, 3.7% (1/27) in twin group and 33% (1/3) in triplet group had history of previous PTB. It is stated that a major risk factor for PTL is prior PTB[16]. The recurrent, familial and racial nature of PTB has led to the suggestion that genetics may play a causal role[17]. Although it had been suggested that the strongest risk factor for PTB is a maternal or fetal genetic predisposition toward PTB,[18] but there were other factors which were more potently associated with PTL in this study.

The other accompanying maternal risk factor was uterine abnormalities including; septate, bicornate and myomatous uterus as well as cervical incompetence. Uterine anomalies are proved to be risk factors for PTB.[2] Cerclage for the treatment of incompetent cervix is the most frequent surgical intervention which was done during pregnancy in this study. Other accompanying maternal medical problems were cardiac and thyroid diseases followed by symptomatic renal stones, as showed in Table 1.

This study showed that preterm ROM happened in 52 (17.5%) of singleton, 12 (44.4%) of twin and 2 (66.6%) of triplet pregnancies during their hospital course. Also thirty singleton (10.1%) and two twin (7.4%) cases suffered from a febrile illness. It is hypothesized that intrauterine infections trigger PTL by activation of immune system and inflammatory cytokines which stimulates the production of prostaglandins which stimulate uterine contractions and preterm rupture of membranes[4][19][20].

It is reported that, in humans, as the number of fetuses per pregnancy increases, the percentage of male conspectuses decreases[21]. However, in this study, 49% (98/200) of singleton neonates, 33.3% (14/42) of twins and 55.5% (5/9) of triplets were female. These results did not show any significant difference in the neonatal sex ratios in the three groups with PSUC.

Logically the number of NICU admitted neonates and duration of their admissions increased with increasing the fetal numbers and decreasing the neonatal birth weights and gestational ages (Table 3). Increasing the rate of neonatal deaths with increasing the fetal numbers are predictably observed in the singleton and twin groups, but not in the triplets. Six of singleton neonates (3%) died in comparison to 4/42 (9.52%) of twins but none of the triplet babies died in this study, which may be contributed to the small sample size.

Birth defects were reported to be associated with preterm birth and low birth weight[22]. Five neonates (2.5%) from the singleton group had congenital anomalies which is consistent with the rate of congenital anomalies in the normal population to be 3%. However, 15.7% of twins and 35% of triplets had congenital malformations. This study confirms the previous reports that the incidence of congenital anomalies is appreciably increased in twin and higher order multiple gestations compared with singletons[21].

Development of preterm regular uterine contractions even in the absence of cervical changes should be considered as a potent risk factor for PTB. The most frequently associated maternal risk factors in this study were history of abortion, infertility and previous PTB, and the most frequently associated complications were preterm ROM, vaginal bleeding and febrile diseases.
